# Biodegradable manganese-doped hydroxyapatite antitumor adjuvant as a promising photo-therapeutic for cancer treatment

**DOI:** 10.3389/fmolb.2022.1085458

**Published:** 2022-11-23

**Authors:** Sumin Park, Jaeyeop Choi, Vu Hoang Minh Doan, Se Hwi O

**Affiliations:** ^1^ Industry 4.0 Convergence Bionics Engineering, Pukyong National University, Busan, South Korea; ^2^ Smart Gym-Based Translational Research Center for Active Senior′s Healthcare, Pukyong National University, Busan, South Korea

**Keywords:** manganese doped hydroxyapatite, photothermal therapy, photodynamic therapy, antitumor adjuvant, cancer treatment

## Abstract

The efficiency of a cancer therapy agent depends on its ability to eliminate tumors without endangering neighboring healthy tissues. In this present study, a novel multifunctional property enriched nanostructured system was synthesized on manganese-doped hydroxyapatite (Mn-HAp) conjugated with counter folic acid (FA) IR-783 fluorescence dye. The tailored synthesis of nano rod-shaped Mn-HAp nanoparticles with high surface area allows to conjugate FA/IR-783 dye which enhanced retention time during *in vivo* circulation. The drug-free Photothermal Photodynamic therapy mediated cancer treatment permits the prevention of collateral damages to non-cancerous cells. The safe HAp biomaterial matrix allows a large number of molecules on its surface due to its active different charge moieties (Ca^2+^/PO_4_^3-^) without any recurrence toxicity. The doped Mn allows releasing of Mn^2+^ ions which triggered the production of toxic hydroxyl radicals (•OH) *via* Fenton or Fenton-like reactions to decompose H_2_O_2_ in the tumor sites. Herein, IR-783 and FA were selected for targeted fluorescence imaging-guided photothermal therapy. 6The PTT performance of synthesized nanostructured system shows enhanced potential with ∼60°C temperature elevation with 0.75 W∙cm^−2^ power irradiated within 7 min of treatment. PDT activity was also observed initially with Methylene Blue (MB) as a targeted material which shows a drastic degradation of MB and further *in vitro* studies with MDA-MB-231 breast cancer cell line show cytotoxicity due to the generated reactive oxygen species (ROS) effect. FA/IR-783 conjugated Mn-HAp nanoparticles (2.0 mol% Mn-HAp/FA-IR-783) show significant tumor-specific targeting and treatment efficiency while intravenously injected in (tail vain) BALB/c nude mice model without any recurrence. The synthesized nanostructured system had ample scope to be a promising Photo-Therapeutic agent for cancer treatment.

## 1 Introduction

A diverse population of cancer cells with various genetic abnormalities make up tumors (Roma-Rodrigues et al., 2020). Tumor-targeted drug delivery systems has grown in popularity as a way to boost the therapeutic effectiveness of anticancer medications and lessen their harmful side effects in living organisms (Bharathiraja et al., 2018; Shi et al., 2020). Traditional intravenous or subcutaneous injections would require the drugs to travel a great distance through the circulatory system to reach the tumor, and blood or body fluids would significantly dilute the drug’s concentration, leading to the metabolism of most pharmaceuticals in the circulatory system (Dewhirst and Secomb, 2017; Qi et al., 2021). The goal of recent decades’ efforts has been the utilization of diverse techniques to treat cancer. One of the most promising methods for better cancer therapy without any side effect is non-chemotherapeutic tumor treatment (Jang et al., 2018; Senapati et al., 2018). Since non-chemotherapeutic tumor treatment has fewer adverse effects than chemotherapy, it has drawn a lot of attention. Nanoparticles’ multifunctionality, site selectivity, and peripheral interaction with targeted molecules offer significant therapeutic benefits (Rosenblum et al., 2018). However, there are still limitations to nanomaterials-based non-chemotherapy, such as poor targeting and limited retention. Photothermal therapy (PTT) is anticipated to make strides in the realm of treating tumors (Santhamoorthy et al., 2021). A technique called photothermal therapy (PTT) uses photothermal materials to absorb near-infrared (NIR) light and turn it into heat to treat malignancies (Santha Moorthy et al., 2018; Wang et al., 2020). NIR light with wavelengths between 700 and 1,300 nm can penetrate tissues well and cause little tissue damage with a penetration depth of up to 10–15 mm (Phan et al., 2018; Wu et al., 2020). The use of PTT alone or in conjunction with Photodynamic therapy (PDT) and multi-mode imaging has emerged as a research hotspot at the moment due to the significant study of various photothermal therapies using various photothermal materials, such as nanometals, nanocarbons, organic preparations, and so on (Shanmugam et al., 2014; Kumar et al., 2015; Hu et al., 2017). An effective method of treating cancer is photodynamic therapy (PDT) (Hou et al., 2019). Reactive oxygen species (ROS), such as peroxides, superoxide, singlet oxygen (^1^O_2_), *etc.*, produced under light irradiation are mostly used to accomplish cell ablation for therapeutic purposes (Wang et al., 2019). Present day imaging guided PTT/PDT synergistic approaches are more efficient and useful for cancer treatment (Manivasagan et al., 2019b). IR-783, an effective near-infrared heptamethine cyanine dye with a potent ability to permeate biological tissues, targets mitochondria without altering tumor-targeting ligands to concentrate close to tumor tissue (Manivasagan et al., 2019a; Li et al., 2019). Among all NIR agents, IR-783 has drawn the most attention due to its superior imaging and tumor targeting capabilities (Deng et al., 2018). Hydroxyapatite (Ca_10_(PO_4_)_6_(OH)_2_, HAp), an inorganic component mostly found in bone and teeth, has strong biological activity and biocompatibility (Kim et al., 2018; Mondal et al., 2018). HAp of various morphologies and surface properties shows structural benefits, highly active surface, distinctive physical and chemical properties, ease of modification, biocompatibility, nontoxic, and anti-inflammatory properties, as drug carriers for the delivery of a variety of pharmaceutical molecules (Neelgund and Oki, 2016; Mondal et al., 2019; Tao et al., 2021). For cell growth and metabolism, folic acid (also known as folate or vitamin B9) is essential. Folate is used as a component for fighting cancer because of its high affinity for the folate receptor proteins. As a tumor biomarker, the folate receptor is overexpressed in specific malignant cells, including those seen in breast, ovarian, lung, kidney, brain, and colon cancers (Jurczyk et al., 2021). Drug uptake by cells through endocytosis is improved by folate-conjugated drug delivery methods (Sabharanjak and Mayor, 2004; Frigerio et al., 2019). Researcher worldwide reporting many potential therapeutic approaches for cancer management, which opens new insights to treat cancer. Till date very few researches reported the potential HAp as a matrix for PTT guided cancer treatment. Recently Chang et al. (2021) reported ROS activated PTT responsive HAp nanoplatforms for anticancer applications. The researcher used HAp@IR-780 nanoparticles for PTT cancer therapy. The study lack of targeted therapeutic approaches. So, the limitation of this study to local intra tumoral application only. In another report Cheng et al. (2021) reported glucose targeted HAp/Indocyanine green hybrid nanoparticles for tumor therapy. The experimental study is limited specially the synthesis part associated with high pH value which might limits the association of functional ligands with HAp nanoparticles. The authors did not report any imaging approach to diagnose the tumor or cancer region.

**FIGURE 1:**
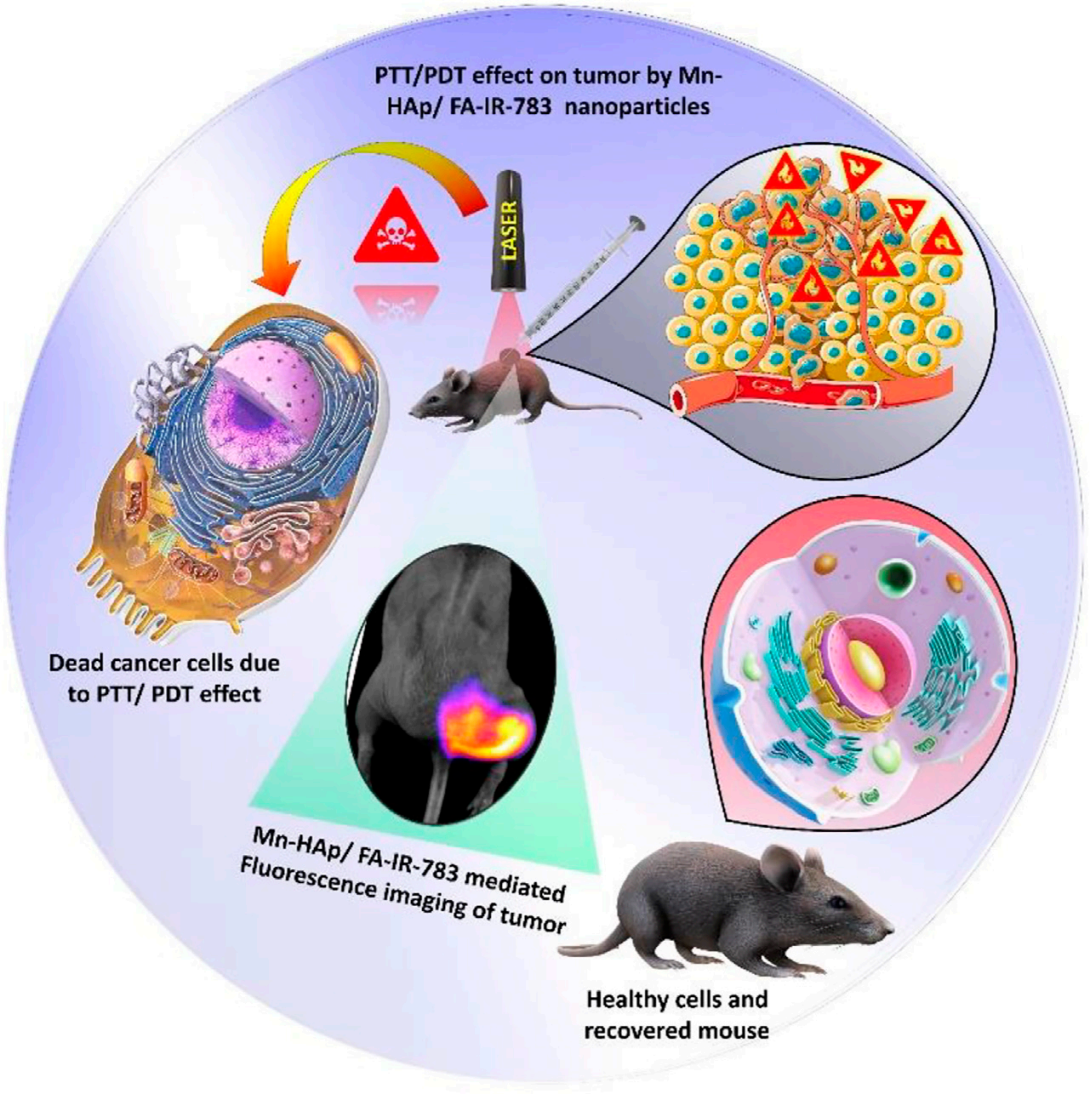
Schematic representation of fluorescence imaging guided Photothermal Photodynamic therapeutic efficacy of Mn-HAp/FA-IR-783 nanoparticles.

In this present study, a novel nanostructured system with enhanced multifunctional properties was created using manganese-doped hydroxyapatite (Mn-HAp) linked with the counter folic acid and IR-783 fluorescence dye. For targeted fluorescence imaging guided photothermal/photodynamic therapy, IR-783 and FA were chosen. *In vitro* cell experiments and *in vivo* systemic efficacy were performed using MDA-MB-231 breast cancer cells and BALB/c tumor-bearing nude mice to assess the biological effects of the synthesized nanoparticles. The synthesized nanoparticles proved its synergistic imaging guided PTT/PDT effect on cancer cells, which could be useful for targeted cancer treatment (Figure 1).

## 2 Experimental

### 2.1 Materials and methods

All of the chemicals and ingredients were purchased from Sigma-Aldrich (St. Louis, United States) and used without further purification. Cell culture Dulbecco’s modified Eagle’s medium (DMEM), antibiotic, trypsin, phosphate buffer saline (PBS), and fetal bovine serum (FBS) were purchased from HyClone (Texas, United States). Deionized (DI) water (*R* = 18.0 M Ω) from the Millipore deionizer was used in the entire experimental study. MDA-MB-231 cells and BAMB/c nude mice (6 weeks, 18–20 g, female) were purchased from Korean Cell Line Bank (Seoul, Republic of Korea) and Hana Biotech (Gyeonggi-do, Republic of Korea), respectively.

#### 2.2.1 Synthesis of manganese doped hydroxyapatite

Mn_x_–HAp nanoparticles are synthesized with Ca(NO_3_)_2_·4H_2_O, (NH_4_)_2_HPO_4_, and Manganese (II) chloride [MnCl2]. First, 175 ml and 125 ml of deionized water were used to dissolve 0.24 M of calcium nitrate tetrahydrate and 0.29 M of diammonium hydrogen phosphate, respectively. An exact amount of manganese (II) chloride was added to 0.24 M of calcium nitrate tetrahydrate solution to synthesize 0.5, 1.0, and 2.0 mol% Mn-HAp. To maintain the pH of all solutions to 9, ammonia solution was added, and next the diammonium hydrogen phosphate solution was added dropwise to calcium nitrate tetrahydrate solution. Set the pH of the combined solution to 9 and stir for 6–8 h at 180 rpm. Deionized water was used to rinse, dry, and then calcine the synthesized product for 1 hour at 600°C from the acquired precipitates (Park et al., 2022).

#### 2.2.2 Synthesis of functional Mn-HAp (Mn-HAp-NH_2_)

The steps involved in preparing HAp-NH_2_ were as follows: A certain amount of ethanol and water were combined (ethanol/H_2_O = 27 ml: 3 ml), and then 100 mg of 3-aminopropyl)-triethoxysilane (APTES) was added. The stirring period was set for 30 min. An amount of 200 mg of HAp was added to the aforementioned hybrid solution, which was then sonicated for 8 min and agitated for 4 h. The pH of the mixture was then raised to around 9–10 with aqueous ammonia solution, and the mixture was then stirred at room temperature for 4 h. The final step involved washing the product 5 times with distilled water before centrifuging it with anhydrous ethanol. The precipitate was then dried to produce HAp-NH_2_ powder while the oven temperature was adjusted to 40–50°C.

#### 2.2.3 Synthesis of Mn-HAp/FA-IR-783

All of the reaction’s steps were completed in complete darkness. Initially 75 mg of HAp-NH_2_ powder with 10 ml of anhydrous DMF solution were mixed and stirring for 15 min, followed by 45 min stirring of 50 μl triethylamine as a catalyst. The reaction solution received 200 g of IR-783. Next 10 mg FA was added to the following mixture and stirring for 24 h at 45°C. The resulting precipitate was thoroughly cleaned before being freeze-dried to create a solid powder.

### 2.3 *In vitro* photothermal efficacy of Mn-HAp/FA-IR-783

25 mg/ml of Mn-HAp/FA-IR-783 was combined with distilled water, dispersed using ultrasonic, and exposed to an 808 nm NIR laser for irradiation. The solution’s temperature rise was monitored every 30 s using an infrared laser and infrared thermal imager. In this experiment, the distilled water group was compared with the Mn-HAp/FA-IR-783 group. The stability of Mn-HAp/FA-IR-783 under laser irradiation was further assessed by irradiating 3 ml of the material (200 μg/ml) for five cycles, with each temperature rise lasting 7 min. Each cycle ended with the sample being cooled to room temperature before the next one started. In the experiment, the temperature variations for each sample during each cycle were noted.

### 2.4 Cell cytotoxicity

To assess the cytotoxicity of *in vitro* cells, the MTT test was performed. In the experiment, DMEM is carefully mixed with 10% FBS and 1% antibody; MDA-MB-231 cells are counted and diluted before being placed on 96-well plates (seeded cell density in each well 1 × 10^4^ cells). The incubator’s temperature was set to 37°C with 95% air and 5% CO_2_. After overnight incubation, the media was mixed with HAp, IR-783 and Mn-HAp/FA-IR-783 (50–250 μg/ml). After a 24-h incubation period, MTT was evenly added to the cells in each well, and they were then treated for 4 h in the dark. To determine the percentage of cell viability, a microplate reader (490 nm) was used to record optical absorbance (OD value). Finally, cell activity was calculated using the following formula:

$$Cell\;viability\;\%={OD}_{sample}\;/{OD}_{control}\times 100\%$$

Where, ${OD}_{sample}$ represents the optical absorbance of cells that had undergone various treatments; and ${OD}_{control}$ represents the optical absorbance of untreated cells.

### 2.5 Cellular uptake of Mn-HAp/FA-IR-783 *in vitro*

The feasibility of ingesting Mn-HAp/FA-IR-783 in cancer cells was studied by CLSM. MDA-MB-231 cells were inoculated into a confocal plate of 1 × 10^4^ cells/well at 37°C for 24 h under the same environment as before, and then the culture medium was changed. After the cells were cultured for 6 h, the media was aspirated. PBS was used to wash cells 3 times. After washing AO/PI Live/Dead cell staining was performed under confocal laser scanning microscopy.

### 2.6 ROS detection of Mn-HAp/FA-IR-783

Methylene Blue (MB) was selected as the target material for the detection of extracellular ROS. Mn-HAp/FA-IR-783 was then added to the 5 mM MB solution. After irradiated with 0.75 W cm^−2^ 808 nm laser every 5 min interval UV absorption of MB was recorded. To study *in vitro* ROS effect, MDA-MB-231 cell lines were seeded in 12 well plates. After culturing for 24 h, the culture media was mixed with HAp, Mn-HAp/FA-IR-783 nanoparticles. Post laser treatment (4 h later), diluted 2′,7′-Dichlorodihydrofluorescein diacetate (DCFH-DA) solution was added. The culture plates were incubated 30 min in CO_2_ incubator. Finally, the cells were washed four times with serum-free culture medium to remove excess DCFH-DA solution, and observed under confocal microscope.

### 2.7 Photothermal therapy of Mn-HAp/FA-IR-783 in MDA-MB-231 tumor nude mouse irradiated by NIR laser

The *in vivo* treatment experiment was initiated when the tumor size in the MDA-MB-231-induced nude mice reached 100–120 mm^3^. The experimental mouse was divided into three groups: Mn-HAp/FA-IR-783 nanoparticles only, Laser only, and Mn-HAp/FA-IR-783 combined with Laser. The nanoparticles were delivered into the mouse’s tail vein using sterile syringes. The dosage was 1.5 mg/kg and was injected into the tail vein of the mouse based on weight. Post 6 h, time period after injection the tumor was irradiated using 808 nm laser. The corresponding PTT images were captured using the infrared thermal camera.

### 2.8 Animal model

All animal testing was done in compliance with institutional rules and regulations for animal care services that were approved by Pukyong National University in Busan, Republic of Korea (PKNUIACUC-2021-25). The female BALB/c naked mice (18.7 ± 0.7 g), aged between six and 7 weeks were procured from Orient Bio Inc. (Seongnam, Republic of Korea). MDA-MB-231 cells (10^6^ cells) suspended in 100 μl of PBS were injected subcutaneously into the flanks of all the mice. The animals were kept under observation with regular care until the tumors reached a volume of ∼100 mm^3^, measured using digital calipers. Five groups of animals were separated. For this experiment, a total of 20 animals were used, as shown in Supplementary Table S1.

The animals with tumors received 100 μl of a 200 μgmL^−1^ Mn-HAp/FA-IR-783 nanoparticle solution through injection. After that, until the active nanoparticles reached the desired tumor site, the mice were kept in a suitable environment and under regular observation. After each 2 h interval the animals were studied under the fluorescence imaging system to identify the tumor region. After 6 h interval sufficient fluorescence signals received from the tumor region. An optical fiber connected to an 808 nm laser diode was used to deliver 7 min of 0.75 W cm^−2^ radiation to each mouse’s tumor. All five groups, which include (I) control group without tumor, (II) tumor bearing mouse without any treatment (III) tumor bearing mouse with laser irradiation only (IV) Mn-HAp/FA-IR-783 nanoparticles injected mouse, and (V) Mn-HAp/FA-IR-783 injected mice with laser irradiation. Before irradiation, a probe thermometer was placed into the center of the tumor. The temperature of the tumor was recorded during the irradiation at 1 s intervals.

### 2.9 Histological analysis

Following the completion of the 3-week treatment, the mice from each group were euthanized, and the tumor as well as the five major organs (kidney, liver, spleen, heart, and lungs) were harvested. The experimental organs were washed in saline water and stored in 10% formalin for fixing and staining. The tissues were immersed for 24 h, preserved with paraffin, and cut into 4 m-thick slices for hematoxylin and eosin (H&E) staining. The histology slides were then seen under a microscope.

### 2.10 Statistical analysis

Each result was presented as a mean standard deviation (SD). Using the program Origin 9.0, all of the charts in this article were produced. Analysis of variance was used to statistically determine whether the experiment’s results were similar (Student t-test). The *p*-value < 0.05 cutoff was used to determine statistical significance.

## 3 Results and discussion

### 3.1 X-ray diffraction

**FIGURE 2:**
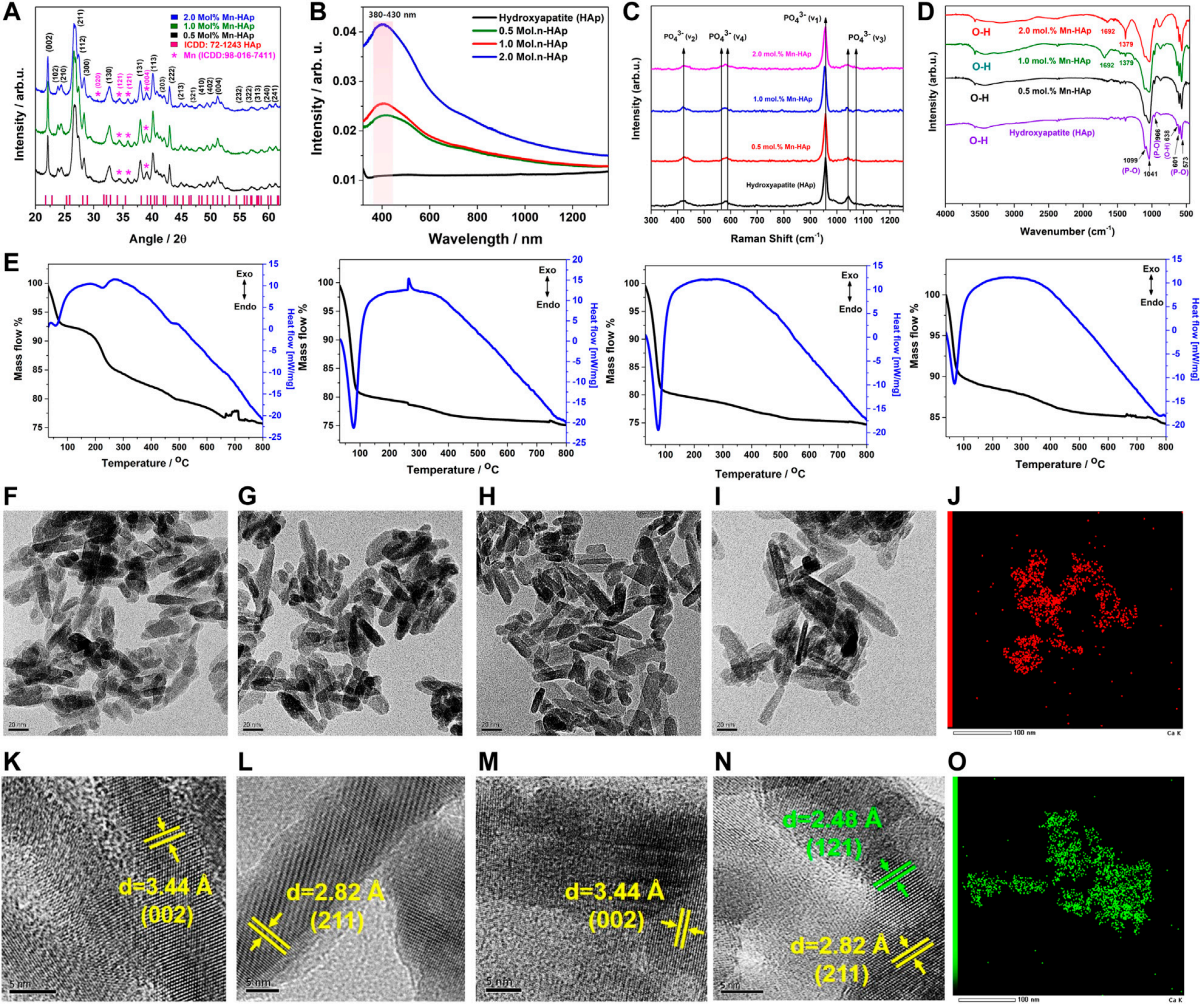
**(A)** XRD analysis of HAp and Mnx-HAp (x = 0.5, 1.0, and 2.0 mol%) nanoparticles **(B)** UV-Vis Diffusive Reflectance Spectroscopy analysis of HAp and Mnx-HAp (x = 0.5, 1.0, and 2.0 mol%) nanoparticles **(C)** Raman shift and **(D)** FTIR analysis of HAp and Mnx-HAp (x = 0.5, 1.0, and 2.0 mol%) nanoparticles **(E)** Thermogravimetric and differential thermal analysis of HAp and Mnx-HAp (x = 0.5, 1.0, and 2.0 mol%) nanoparticles **(F–I)** FE-TEM study of HAp and Mnx-HAp (x = 0.5, 1.0, and 2.0 mol%) nanoparticles and **(K–N)** their corresponding HR-TEM analysis with inter atomic distance measurement **(J)** EDS mapping for “Mn” **(O)** EDS mapping for “Ca”.

The XRD analysis of HAp and Mn-HAp (Mn: 0.5, 1.0, 2.0 mol%) nanoparticles were studied and compared with the standard JCPDS (# 00-009-0432) data of the pristine HAp (Figure 2A). Sharp peaks on XRD patterns show that the calcined HAp nanoparticle has good crystallinity. The maximum intensity peak was found at 2θ value of 31.7°, corresponding to (211) planes for pristine HAp. The diffraction peaks for HAp at 25.5°, 28.6°, 32.3°, 39.2°, 46.4°, 49.1°, 53.4°, 61.3°, and 64.1°, respectively, indicate the computed (002), (210), (112), (130), (222), (213), (004), (214), and (304) planes (2 diffraction angle). Debye Scherer’s relation was used to determine the average crystallite size of the HAp particles over the peak with the highest intensity (211):

D=0.9λ /β⁡cos⁡θ

where D stands for average crystallite size, FWHM (full width at half maximum) of the peak, diffraction wavelength (0.154059 nm), and diffraction angle are all mentioned. Table 1 shows the typical crystallite size of calcined HAp and various Mn-HAp (Mn: 0.5, 1.0, 2.0 mol%).

**TABLE 1:** Typical crystallite size calculation of HAp and Mn-HAp (Mn: 0.5, 1.0, 2.0 mol%).

Sl. No.	Sample	Plane (211) FWHM	Average single crystal size D (nm)
1	Hydroxyapatite (HAp)	0.31	27.8
2	0.5 mol% Mn-HAp	0.32	27.4
3	1.0 mol% Mn-HAp	0.36	24.3
4	2.0 mol% Mn-HAp	0.39	21.7

### 3.2 UV-visible diffusive reflectance spectroscopy analysis

The synthesized Mn-HAp nanoparticles were subjected to diffuse reflectance spectroscopy, in which the valence electrons are excited to empty orbitals using visible light. Figure 2B shows the UV-vis DRS absorption spectra of produced HAp and Mnx-HAp nanoparticles (x = 0.5, 1.0, and 2.0 mol%). There are no significant absorption peaks found for HAp material alone. Whereas a distinct broad absorption band was found for materials containing varying amounts of Mnx-HAp (x = 0.5, 1.0, and 2.0 mol%) (in the range of approximately 380–430 nm). This investigation supports the successful doping of manganese on the HAp matrix.

### 3.3 Thermogravimetric differential thermal analysis

TG-DT analysis was used to determine the thermal stability of HAp and Mnx-HAp nanoparticles (x = 0.5, 1.0, and 2.0 mol%) using an alumina crucible heated to 800°C at a heating rate of 10°C/min (Figure 2E). Pure HAp has a mass loss of about ∼22.65%. Contrarily, mass losses of ∼21.75%, ∼19.89%, and ∼12.73% were found for the other samples Mnx-HAp nanoparticles (x = 0.5, 1.0, and 2.0 mol%), which are comparably lower than HAp. The doping of Mn constantly increases the thermal stability.

Additionally, the differential thermal analysis curve for HAp and Mnx-HAp nanoparticles (x = 0.5, 1.0, and 2.0 mol%) are represented in Figure 2E. The differential thermal curves of each Mn-HAp sample have a similar shape at temperatures between approximately 55 and 515°C, and the HAp graph exhibits a similar sort of curve at temperatures between 125 and 425°C. There were, however, no discernible exothermic or endothermic processes. The TG-DTA graph supports the Mn-HAp nanoparticles’ heat stability.

### 3.4 Transmission electron microscopy analysis

The ultrastructure and their corresponding TEM and EDS analysis of the synthesized HAp and Mnx–HAp (x = 0.5, 1.0, and 2.0 mol%) nanoparticles is represented in Figures 2F–O. The synthesized nanoparticles show elongated rod-shaped structure with average length of ∼26 ± 2.8 nm for pristine HAp (Figure 2F), ∼26 ± 3.2 nm for 0.5 mol% Mn-HAp (Figure 2G), ∼27 ± 3.1 nm for 1.0 mol% Mn-HAp(Figure 2H, ∼28 ± 2.1 nm for 2.0 mol% Mn-HAp (Figure 2I) nanoparticles. The HR TEM study revealed an atomic distance of 3.4 Å (002), 2.8 Å (211), 3.4 Å (002), 2.8 Å (211) which corresponds to the hydroxyapatite plane for HAp and Mnx–HAp (x = 0.5, 1.0, and 2.0 mol%) nanoparticles respectively (Figures 2K–N). Figure 2N, shows the atomic distance of 2.4 Å (121) associated to the Mn plane. The elemental analysis confirmed the presence of “Ca” and “P” due to HAp and “Mn” due to doping materials (Figures 2J and O). The detailed EDS analysis is represented in supplementary section (Please see the supplementary data Supplementary Figure S1).

### 3.5 FTIR spectroscopy analysis

The synthesized HAp and Mnx-HAp (x = 0.5, 1.0, and 2.0 mol%) nanoparticles, shown in Figure 2D, were examined using FTIR spectroscopy. The nanoparticles exhibit a variety of peaks in their FTIR spectra, which are described as follows. In all samples, the bands at 966–1,099 cm^−1^ and 573–601 cm^−1^ are related to the P-O bond caused by phosphate groups (Vinoth Kumar et al., 2021). The phosphate group’s v3 stretching mode is responsible for the peak at 1,041 cm^−1^. Peak P-O tension ν1 stretching took place at 966 cm^−1^ (Park et al., 2022). The v4 bending vibration based on the phosphate group is shown in the peak at 573 cm^−1^. The band found between 3,570 cm^−1^ and 638 cm^−1^ demonstrated the hydroxyl group (O-H) correlating to the O-H stretching of HAp (Prasanna and Venkatasubbu, 2018). Additionally, the peaks for the phosphate group and characteristic vibration in produced nanoparticles are unaltered.

### 3.6 Raman spectroscopy analysis

In order to investigate the HAp and Mnx-HAp nanoparticles (x = 0.5, 1.0, and 2.0 mol%), detailed chemical structure, phase, and molecular interactions are studied using the effective non-destructive method of Raman spectroscopy. HAp and Mnx-HAp nanoparticles (x = 0.5, 1.0, and 2.0 mol%) functional groups, bands, and modes’ Raman spectra are shown in Figure 2C. Both samples displayed similar functional groups and modes to the HAp and Mnx-HAp nanoparticles (x = 0.5, 1.0, and 2.0 mol%). The *ʋ1*(PO_4_) band at 962 cm^−1^ is very intense which is exactly similar with HAp and all the different concentration Mn doped HAp nanoparticles. The Raman active modes were detected at 1,033 cm^−1^, 1,046 cm^−1^, 1,076 cm^−1^ (*ʋ3*(PO_4_)), 502 cm^−1^, 581 cm^−1^, 592 cm^−1^ (*ʋ4*(PO_4_)), and 432 cm^−1^, 447 cm^−1^ (*ʋ2*(PO_4_)), respectively (Yilmaz and Evis, 2014). However, when Mn was doped on HAp nanostructures, peak locations were changed. Additionally, there is a strong correlation between the FTIR data and the peak positions determined by the functional groups.

### 3.7 Photodynamic performance evaluation of Mn-HAp/FA-IR-783

**FIGURE 3:**
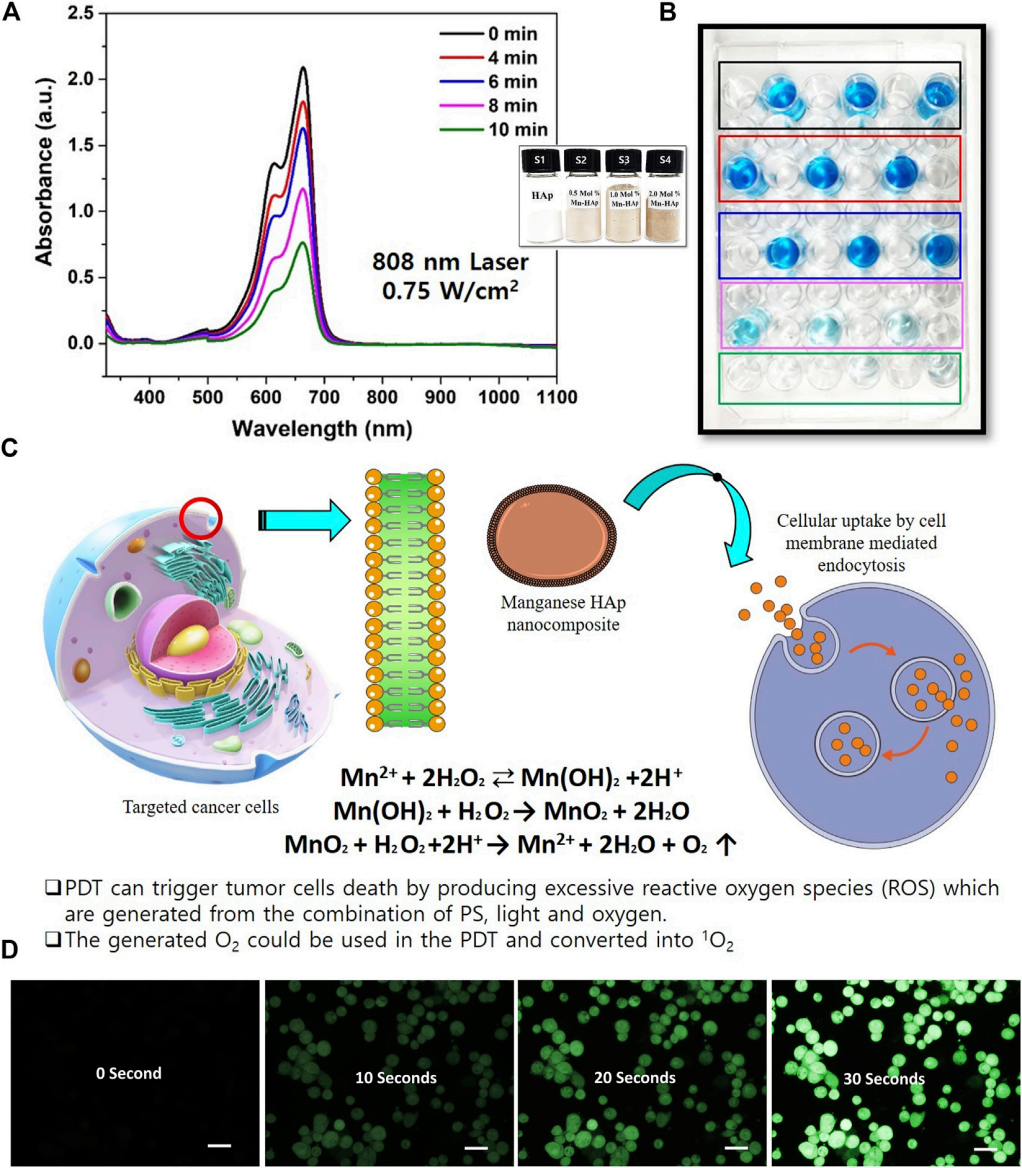
Photodynamic effect of Mn-HAp nanocomposites. **(A)** UV–vis absorption spectra of Methylene Blue (MB) mixed with HAp and Mnx-HAp/FA-IR-783 (x = 0.5, 1.0, and 2.0 mol%) nanoparticles treatments (Inset: synthesized HAp and Mnx-HAp (x = 0.5, 1.0, and 2.0 mol%) nanoparticles and) **(B)** PDT effect on MB solution **(C)** The mechanism of cellular ROS generation due to manganese ions **(D)** Detection of O_2_ production due to the PDT effect (reaction with DCFH-DA) of synthesized nanoparticles (each scale bar is 20 micro meter).

PDT can cause tumor cells to die by overproducing reactive oxygen species (ROS), which are created when photosensitizer (PS), light, and oxygen combine. Mn^2+^ may be transformed into Mn(OH)_2_ in the presence of H_2_O_2_ in a neutral environment, and the unstable Mn(OH)_2_ could subsequently be oxidized by H_2_O_2_ to produce MnO_2_, according to previous Mn^2+^ studies (Fu et al., 2019; Liang et al., 2022). The catalytic breakdown of H_2_O_2_ to produce oxygen and simultaneously reduce to Mn^2+^ can be strongly induced by the created MnO_2_ (Figure 3C). The following reactions show the entire process:

$${Mn}^{2+}+2\;H_{2}O_{2}\;⇄\;Mn{\left(OH\right)}_{2}+2H^{+}$$

$$Mn{\left(OH\right)}_{2\;}+H_{2}O_{2}\;\to \;{MnO}_{2\;}+2H_{2}O$$

$${MnO}_{2}+H_{2}O_{2}+2H^{+}\;\to \;{Mn}^{2+}+2H_{2}O+O_{2}\;↑$$

In this present study HAp and Mnx-HAp/FA-IR-783 (x = 0.5, 1.0, and 2.0 mol%) nanoparticles were synthesized rod shaped with a high surface area (Please see the supplementary section for BET surface area analysis, Supplementary Figure S2). The synthesized nanoparticles further studied for PDT efficiency study (Figures 3A,b1,b2). Here, Methylene blue dye was chosen as targeted dye. After inoculation of different concentration nanoparticles, the solution was irradiated under different light wavelengths. After optimization 2.0 mol% Mn-HAp/FA-IR-783 nanoparticles were used for *in vitro* and *in vivo* study. *In vitro* ROS activity was monitored with MDA-MB-231 cell lines incubated with 2.0 mol% Mn-HAp/FA-IR-783 nanoparticles. The culture media was combined with HAp, Mn-HAp/FA-IR-783 nanoparticles after being cultured for 24 h. Post laser treatment (4 h later), diluted 2′,7′-Dichlorodihydrofluorescein diacetate (DCFH-DA) solution was added. The amount of solution used completely covers the acquired cells. After maintaining the same condition, the cells were incubated for 30 min. In order to remove excess DCFH-DA solution, the cells were washed three times with serum-free growth media before being examined under a confocal laser scanning microscope (Figure 3D). After treated with laser the cells emit green fluorescence due to the generation of singlet oxygen ^1^O_2_. This experimental study confirms the PDT efficiency of synthesized 2.0 mol% Mn-HAp/FA-IR-783 nanoparticles.

### 3.8 MTT assay for *in vitro* cytotoxicity study

**FIGURE 4:**
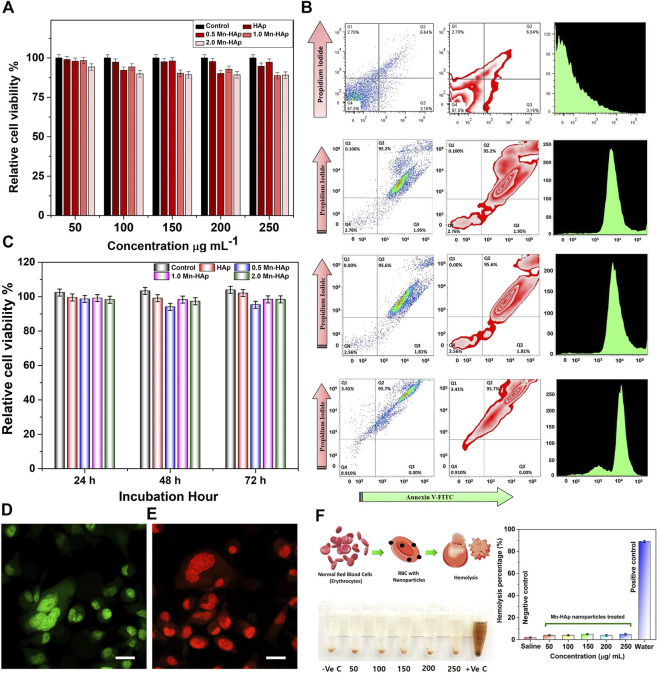
**(A)** MTT assay of HAp and Mnx-HAp/FA-IR-783 (x = 0.5, 1.0, and 2.0 mol%) nanoparticles at different concentration (50–250 μg/ml) with MDA-MB-231 breast cancer cell line. **(B)** FACS study of HAp, 2.0 mol %Mn-HAp/FA-IR-783 (0.5 W cm^−2^, 0.75 W cm^−2^, and 1.0 W cm^−2^) post PTT/PDT treatment effect on MDA-MB-231 cell line. Data were presented as mean ± S.D. (n = 5, *significant *p* < 0.05 compared to control, as statistically significant) **(C)** MTT assay of HAp and Mnx-HAp/FA-IR-783 (x = 0.5, 1.0, and 2.0 mol%) nanoparticles with different time interval (24 h, 48 h, and 72 h) with MDA-MB-231 breast cancer cell line. **(D)** Confocal Laser scanning microscopy of AO/PI stained control (green fluorescent without any nanoparticle) and **(E)** post PTT/PDT treated MDA-MB-231 (dead cells red fluorescence) cell line (each scale bar represents 20 μm)**. (F)** Hemolysis study of 2.0 mol% Mn-HAp/FA-IR-783 nanoparticles with different concentration (50–250 μg mL^−1^).

After 24 h of incubation with various concentrations, the MTT assay of HAp and Mnx-HAp/FA-IR-783 (x = 0.5, 1.0, and 2.0 mol%) nanoparticles exhibited non-toxic properties to the MDA-MB-231 cell line (Figure 4A). More than 90% of the cells remained alive at the lowest dose (50 μg∙mL^−1^), whereas a greater concentration of 250 μg∙mL^−1^ resulted in somewhat higher toxicity which results ∼17% dead cells. As the highest concentration (250 μg mL^−1^) likewise produced ∼82% cell viability, results HAp and Mnx-HAp/FA-IR-783 nanoparticles (x = 0.5, 1.0, and 2.0 mol%) exhibit very minimal toxic behavior up to this concentration. A similar investigation was carried out using 2.0 mol% Mn-HAp/FA-IR-783 nanoparticles, incubated with 200 μg∙mL^−1^ concentrations for 24 h, 48 h, and 72 h with the MDA-MB-231 cell line. When the Mn-HAp/FA-IR-783 concentration reaches 250 μg∙mL^−1^, little toxicity was observed (overall cell viability is ∼85%). Finally, the selected concentration of 200 μg∙mL^−1^ showed ∼86% cell viability (Figures 4A,C). The MTT study further extended with 200 μg mL^−1^ dose concentration of 2.0 mol% Mn-HAp/FA-IR-783 nanoparticles irradiated with variable laser power (0.5 W cm^−2^, 0.75 W cm^−2^, and 1.0 W cm^−2^) (Figure 4C). The control group (without any nanoparticles) shows the highest cell viability, whereas only HAp also shows more than ∼90% viability while irradiated with different laser power densities. The MTT results confirmed all the Mn_x_-HAp/FA-IR-783 (x = 0.5, 1.0, and 2.0 mol%) are lethal while irradiated even with minimum power laser (0.5 W∙cm^−2^). Approximately 95% cells were dead when 0.75 W∙cm^−2^ laser power were used. With increasing laser power intensity, the materials become more lethal. In addition, control group and post PTT/PDT treated MDA-MB-231 cell line groups were assessed by acridine orange/propidium iodide (AO/PI) staining under confocal laser scanning microscopy (Figure 4D, E). To confirm the effect of different laser power density Flow cytometry study was performed.

### 3.9 Flow cytometry study

The flow cytometry was carried out with HAp and 2.0 mol% Mn-HAp/FA-IR-783 nanoparticles treated MDA-MB-231 cell line irradiated with 0.75 W cm^−2^ laser (Figure 4B). The only HAp nanoparticles and laser exposure present in the study of control cells. For the control group, the flow cytometry investigation showed ∼87.5% cells in healthy condition followed by ∼3.16% in the early and ∼6.64% in late apoptosis stage (very less amount ∼2.70% are in necrosis stage due to may be normal cell death). For 2.0 mol% Mn-HAp/FA-IR-783 nanoparticles irradiated with 0.5 W cm^−2^ laser (for 7 min) treated cells shows ∼95.2% cells in late apoptosis condition with ∼1.95% in early apoptosis phase. Very little amount (∼0.1%) was observed in necrosis stage, whereas ∼2.76% cells still shows healthy behavior. With increasing laser power intensity of 0.75 W cm^−2^ the treated cells show ∼95.6% in late apoptosis, ∼1.81% in early apoptosis, and remaining very few cells (∼2.56%) shows viability. While using 1.0 W cm^−2^ laser-irradiation, only ∼0.91% cells were found to be healthy with ∼95.7% in late apoptosis phase, and remaining ∼3.41% in necrosis stage. The FACS study is well corelated with MTT assay which confirms the nanoparticles reproducible performance (Figures 4B,C).

### 3.10 Hemolysis study

Heparin-stabilized blood samples collected from BALB/c nude mice and further extracted by centrifugation were used for the hemocompatibility assay. Red blood cells (RBCs) were collected and diluted with 10X PBS buffer. Various quantities of 2.0 mol% Mn-HAp/FA-IR-783 nanoparticles were added to the RBC suspension (500 μl) at 50, 100, 150, 200, and 250 μg/ml, with medical saline solution served as the negative control and distilled water as the positive control, respectively. All of the solutions were vortexed, allowed to incubated for 4 h at 37°C, and then centrifuged for 5 min at 5,000 rpm. Each sample’s absorbance was measured at a wavelength of 541 nm using a Tecan Infinite F50 microplate reader. The following equation was used to calculate the hemolysis of RBCs in each sample:

$$Hemolysis\;\left(\%\right)=\frac{{Abs}_{Sample}-{Abs}_{Negative\;control}}{{Abs}_{Positive\;control}-{Abs}_{Negative\;control}}\;$$

2.0 mol% Mn-HAp/FA-IR-783 nanoparticles were injected intra venous tail vein region for *in vivo* cancer therapy. Therefore, it is crucial to look into whether the injected nanoparticle is compatible with blood or not. As shown in Figure 4F, 2.0 mol% Mn-HAp/FA-IR-783 demonstrated outstanding blood compatibility with very less hemolytic activities of ∼5.67 ± 0.14% even at a high concentration of 250 μg mL^−1^.

### 3.11 Fluorescence imaging

**FIGURE 5:**
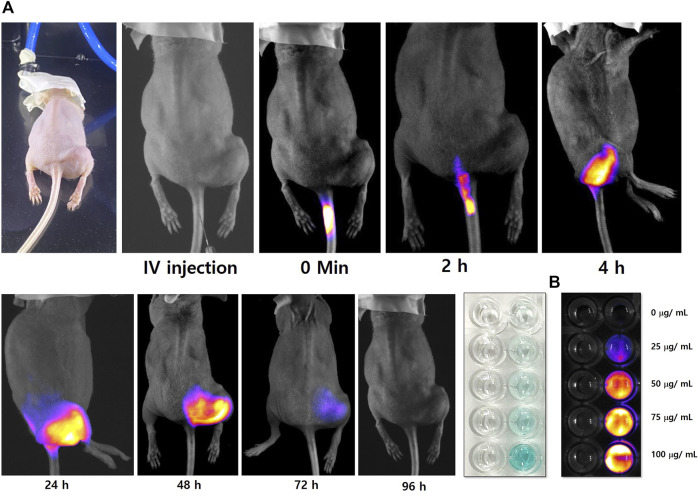
**(A)** *In vivo* tumor targeted fluorescence imaging study of 2.0 mol% Mn-HAp/FA-IR-783 nanoparticles **(B)** *In vitro* study of 2.0 mol% Mn-HAp/FA-IR-783 nanoparticles.

The florescence imaging study was performed by indigenously developed LUX 4.0 imaging system (LUX 4.0; OHLABS, Busan, Republic of Korea). *In vitro* fluorescence imaging study was performed with 2.0 mol% Mn-HAp/FA-IR-783 nanoparticles incubated with MDA-MB-231 cell lines. First, MDA-MB-231 cell lines were left incubated for 24 h for culture plate attachment. Next different concentration (25, 50, 75, 100 μg/ml) prepared nanoparticles were inoculated to the nanoparticles for 4 h. Post incubation the 96 well plates were imaged under LUX 4.0 imaging system (Figure 5B). The study extended with *in vivo* BALB/c mouse model. The tail vein was used to administer 100 μl of 2.0 mol% Mn-HAp/FA-IR-783 (100 μg/ml) to the tumor-bearing nude mice, thereafter fluorescence monitoring (each 2 h interval up to first 12 h, followed by each 24 h interval) was performed using LUX 4.0 imaging system (Figure 5A). The nanoparticle shows excellent fluorescence property under imaging system. The interesting *in vivo* study revealed the folic acid conjugated nanoparticles excellent targeting specifically to the tumor region. In this study Group IV animals (only nanoparticles injection) were selected for the imaging purposes. The injected 2.0 mol% Mn-HAp/FA-IR-783 have excellent targeted imaging capabilities could be a promising feature for clinical level imaging guided cancer therapy.

### 3.12 *In vivo* photothermal therapy

**FIGURE 6:**
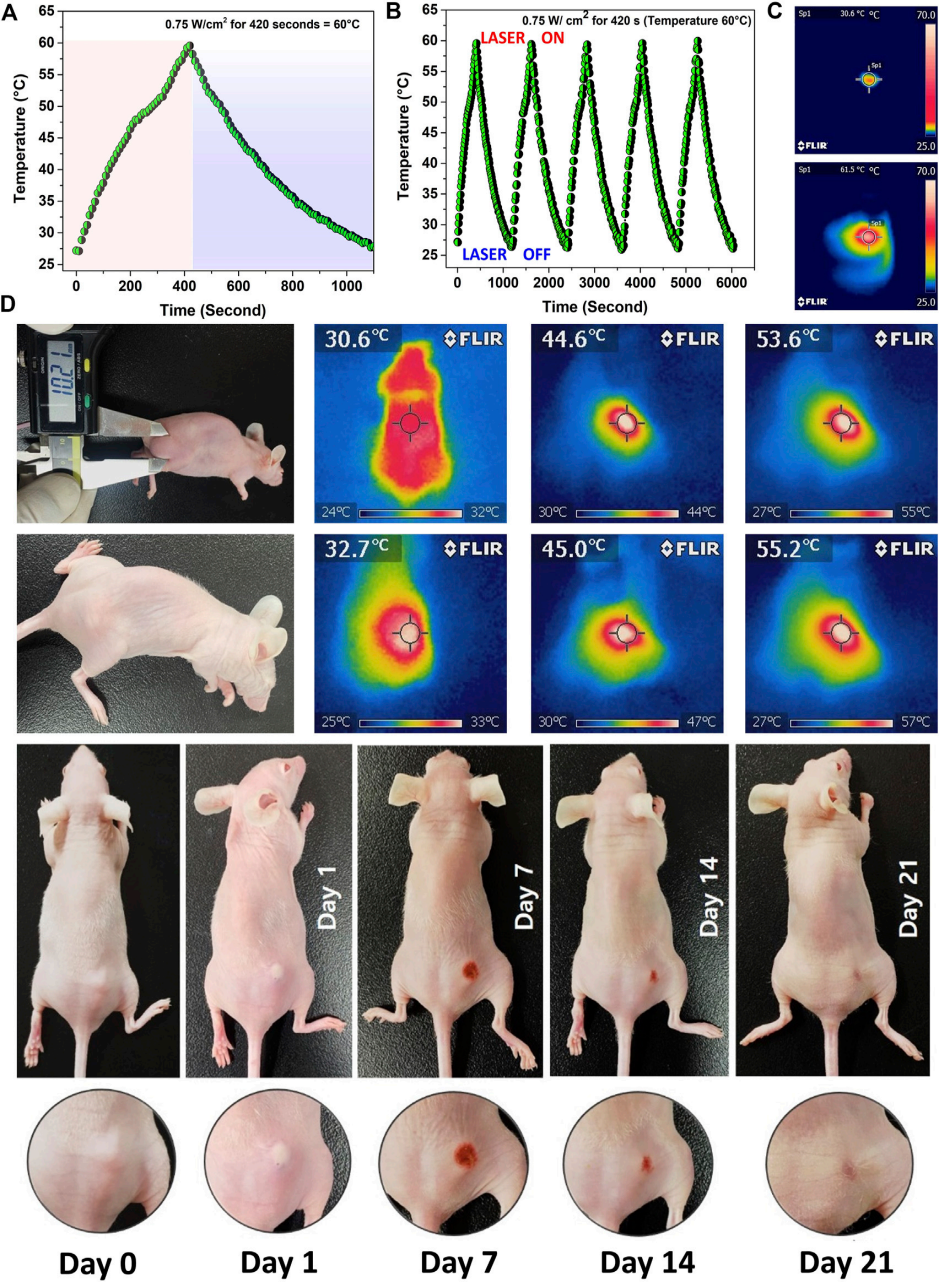
**(A)** Photothermal efficiency measurement of 2.0 mol% Mn-HAp/FA-IR-783 nanoparticles irradiated with a 0.75 W cm^−2^ laser (808 nm) for 7 min **(B)** Laser ON/OFF study for 5 cycles to determine the nanoparticles performance stability **(C)** IR images of initial and final PTT treatment **(D)** PTT/PDT treatment of MDA-MB-231 tumor bearing BALB/c nude mice and their corresponding IR images.

The PTT experiment was started by exposing the tumors to a 0.75 W cm^−2^ laser (808 nm) irradiation for 7 min. The performance of the nanoparticle is shown in Figure 6A. PTT stability of Mn-HAp/FA-IR-783 under repeated five cycles of 808 nm laser (0.75 W.cm-2; ON/OFF) (Figure 6B). The test mice were split into five groups, of which groups III and V received laser therapy. Furthermore, no nanoparticles were administered *via* injection to the mice in groups I, II, or III. Since no heat generation occurred in the absence of nanoparticles, it was confirmed by the *in vitro* study that PTT was ineffective for groups I, II, and III. No laser irradiation was used on groups I or II. The initial experimental investigation began at 25 ± 2°C, although the mouse’s body temperature was 30.6 ± 1.2°C at the beginning. The animals in group II demonstrated a maximum temperature increase of 1.6°C after being exposed to a 0.75 W cm^−2^ laser (808 nm) for 7 min, resulting in a final body temperature of 32.2 ± 0.7°C. The laser dosage alone was unable to raise the temperature because there were no nanoparticles present inside the target tumor location (Mondal et al., 2022). Intratumorally injected with 100 μl of a 200 μg/ml 2.0 mol% Mn-HAp/FA-IR-783 solution, Group V animals were then subjected for 7 min to a 0.75 W cm^−2^ laser (808 nm). According to Figure 6C, IR thermal images, the photothermal treatment causes the mouse’s body temperature to rise from 30.6 to 55.2°C. After the photothermal treatment, the tumor area once more recorded a temperature of 31.4°C, which is close to the mouse’s normal body temperature. The calculated photothermal conversion efficiency of 2.0 mol% Mn-HAp/FA-IR-783 to be *η* ≈ 31.2% which is effective for sufficient heat generation (Please see supplementary information, Supplementary Figure S3). The tumor region’s temperature increase was more than enough to effectively ablate the tumors *in vivo.* This therapy’s safety is confirmed by the fact that no animals died after receiving treatment. Aside from that, the photothermal shock had no discernible effects on the mice’s behavior or body weight. Our investigation found no discernible toxicity or deadly effect, supporting earlier reports’ findings (Terentyuk et al., 2010). All of the mice were monitored for a full 3 weeks following treatment to ensure that the photothermal effects had fully healed (Figure 6D).

### 3.13 Histological analysis

**FIGURE 7:**
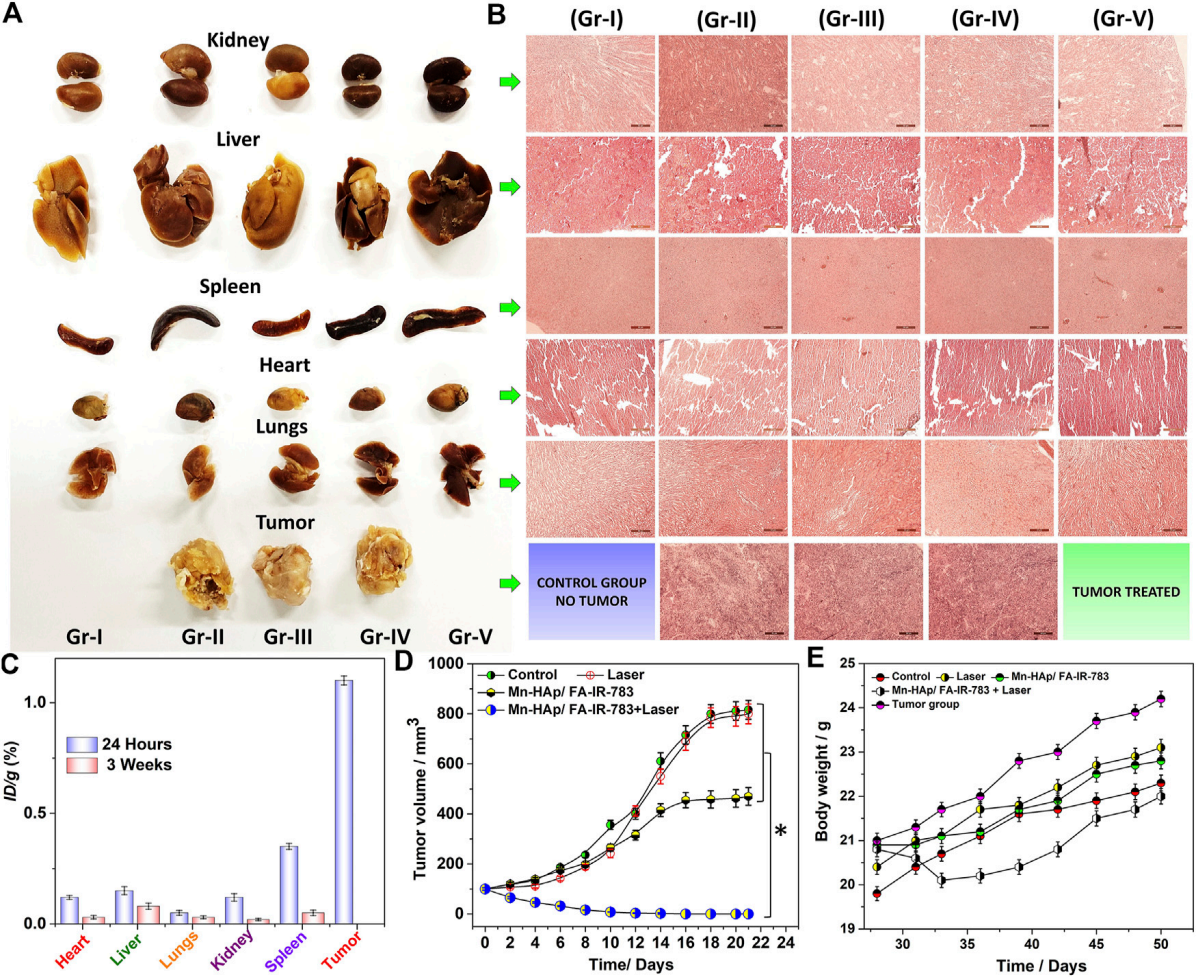
**(A)** Harvested organs (Kidney, Liver, Spleen, Heart, Lungs, Tumor) from the experimental animals **(B)** H and E stained organs imaged under microscope (each scale bar is 50 micro meter) **(C)** Biodistribution of “Mn” in the harvested organs (Kidney, Liver, Spleen, Heart, Lungs) and tumor of the mice treated with 2.0 mol% Mn-HAp/FA-IR-783 nanoparticles for 24 h and 4 weeks post treatment. **(D)** Tumor volume in different groups following PTT/PDT therapies. Data were represented as mean ± S.D. (*n* = 3, *significant *p* < 0.05) **(E)** after PTT treatments, the mice’s body weight.

From tumor induction to photothermal treatment, the *in vivo* experimental study was carried out with a total time period of 7 weeks (Figure 7A). After PTT therapy, all of the mice were monitored and cared for 3 weeks, until full recovery. After the study post-PTT surveillance was completed, the histological examination was carried out. The primary organs were removed and prepared for Hematoxylin and eosin staining as described in the experimental part, including the kidney, liver, heart, spleen, and lungs (Figures 7A,B). After that, an optical microscope was used to look for any physiological and structural alterations in the organs and the tumor. The morphologies of all the cross-sectional tissues appeared to be in good health, and no such abnormalities were seen in any of the organs. Additionally, no discernible color change was seen, therefore all experimental groups’ organs looked to be normal (I-V). Figure 7B displays the tissues’ histological images. The general harmless character of 2.0 mol% Mn-HAp/FA-IR-783 therapies is well supported by the absence of tissue damage, aberrant behavior, and body weight changes. While examining the tumor tissues’ histological section, we noticed several substantial PTT-related alterations. In contrast to the untreated tumors (groups II and IV), which have poorly differentiated cells with granulated deep stained nuclei, the tumors in the 2.0 mol% Mn-HAp/FA-IR-783 treated animals (group III) display medium sized tumors with deep stained cells. Finally, animals treated with 2.0 mol% Mn-HAp/FA-IR-783 and laser irradiation (group V) are completely healed without any tumor recurrence. There were no malignant tissues found in the treated animals of group V when the post-treatment surrounding tissues were collected, only healthy cells with distinct nuclei were observed. All tumor volumes and animal body mass were measured throughout the monitoring period at regular intervals (Figures 7D,E).

### 3.14 Biodistribution of “Mn” (Mn-HAp/FA-IR-783) nanoparticles

At 24-h and 3-week intervals, mice from treatment groups were euthanized in order to study the accumulation of Mn-HAp/FA-IR-783 in organs. The organs underwent an 8-h HNO_3_ treatment after being dissected. Inductively coupled plasma mass spectrometry was utilized to measure the Mn concentration in each group (ICP-MS). The findings demonstrate a significant level of Mn, post 24 h tumor treatment (Figure 7C). At 24 h, the estimated cumulative post-injection dose per gram of tumor tissue was around 1.2% ID∙g^−1^. Due to intravenous injection, the body’s various organs were accumulating the nanoparticles after they mixed with blood flow. The spleen therefore accumulated the most (∼0.28% ID∙g^−1^ at 24 h and ∼0.07% ID∙g^−1^ after 3 weeks), while the kidney, liver, lungs, and heart accumulated, respectively 0.21, 0.21, 0.10, and 0.08% ID∙g^−1^ 24 h post treatment. After 3 weeks, a biodistribution analysis showed that the nanoparticles had been significantly cleared from the body systems, with extremely minute levels found in the spleen, kidney, liver, lungs, and heart, respectively: 0.04, 0.02, 0.05, 0.01, and 0.02% ID∙g^−1^. There were no hazardous or fatal effects as a result of the extremely low nanoparticle concentration inside the organs. The study as a whole concludes that the doses of nanoparticles used here are harmless, nonlethal, and extremely effective for PTT/PDT cancer therapy.

## 4 Conclusion

The management of cancer has been one of the most difficult problems in biomedicine. Today, photothermal and photodynamic therapy are receiving more attention as a potential replacement for chemotherapy and radiotherapy, which have been linked to severe adverse effects. A drug-free synthesized Mn-HAp/FA-IR-783 nanostructured system demonstrating good contrast efficiency with improved PTT/PDT efficacy against tumor bearing mouse model. The Mn-HAp/FA-IR-783 nanoparticles ensembled with broad near-infrared absorbance qualities as well as excellent biosafety, biocompatibility, and photostability. The *in vitro* and *in vivo* experimental work demonstrated potent anticancer efficacy with fluorescence imaging guided PTT/PDT treatment efficacy. PTT uses ablation agents that may turn light into heat whereas combined PDT therapy generates ROS to kill the cancer cells. In terms of fluorescence guided treatment of *in vivo* cancer model, a well-researched standardized dose of 200 μg/ml, 2.0 mol% Mn-HAp/FA-IR-783 nanoparticles with 0.75 W cm^−2^ laser irradiation for 7 min was found to be an efficient and optimal target specific theranostic approach. Researchers may benefit from using this suggested experimental approach to better understand the potential uses of Mn-HAp/FA-IR-783 nanoparticles in future nano-biomedical research.

## Data Availability

The original contributions presented in the study are included in the article/Supplementary Material, further inquiries can be directed to the corresponding author.

## References

[B1] Bharathiraja S. Bui N. Q. Manivasagan P. Moorthy M. S. Mondal S. Seo H. (2018). Multimodal tumor-homing chitosan oligosaccharide-coated biocompatible palladium nanoparticles for photo-based imaging and therapy. Sci. Rep. 8, 500. 10.1038/s41598-017-18966-8 29323212 PMC5764953

[B2] Chang L. Huang S. Zhao X. Hu Y. Ren X. Mei X. (2021). Preparation of ROS active and photothermal responsive hydroxyapatite nanoplatforms for anticancer therapy. Mat. Sci. Eng. C Mat. Biol. Appl. 125, 112098. 10.1016/j.msec.2021.112098 33965108

[B3] Cheng X. Xu Y. Zhang Y. Jia C. Wei B. Hu T. (2021). Glucose-targeted hydroxyapatite/indocyanine green hybrid nanoparticles for collaborative tumor therapy. ACS Appl. Mat. Interfaces 13, 37665–37679. 10.1021/acsami.1c09852 34342216

[B4] Deng G. Li S. Sun Z. Li W. Zhou L. Zhang J. (2018). Near-infrared fluorescence imaging in the largely unexplored window of 900-1, 000 nm. Theranostics 8, 4116–4128. 10.7150/thno.26539 30128040 PMC6096386

[B5] Dewhirst M. W. Secomb T. W. (2017). Transport of drugs from blood vessels to tumour tissue. Nat. Rev. Cancer 17, 738–750. 10.1038/nrc.2017.93 29123246 PMC6371795

[B6] Frigerio B. Bizzoni C. Jansen G. Leamon C. P. Peters G. J. Low P. S. (2019). Folate receptors and transporters: Biological role and diagnostic/therapeutic targets in cancer and other diseases. J. Exp. Clin. Cancer Res. 38, 125. 10.1186/s13046-019-1123-1 30867007 PMC6417013

[B7] Fu L.-H. Hu Y.-R. Qi C. He T. Jiang S. Jiang C. (2019). Biodegradable manganese-doped calcium phosphate nanotheranostics for traceable cascade reaction-enhanced anti-tumor therapy. ACS Nano 13, 13985–13994. 10.1021/acsnano.9b05836 31833366

[B8] Hou H. Huang X. Wei G. Xu F. Wang Y. Zhou S. (2019). Fenton reaction-assisted photodynamic therapy for cancer with multifunctional magnetic nanoparticles. ACS Appl. Mat. Interfaces 11, 29579–29592. 10.1021/acsami.9b09671 31359756

[B9] Hu F. Yuan Y. Mao D. Wu W. Liu B. (2017). Smart activatable and traceable dual-prodrug for image-guided combination photodynamic and chemo-therapy. Biomaterials 144, 53–59. 10.1016/j.biomaterials.2017.08.018 28823843

[B10] Jang B. Moorthy M. S. Manivasagan P. Xu L. Song K. Lee K. D. (2018). Fucoidan-coated CuS nanoparticles for chemo-and photothermal therapy against cancer. Oncotarget 9, 12649–12661. 10.18632/oncotarget.23898 29560098 PMC5849162

[B11] Jurczyk M. Jelonek K. Musiał-Kulik M. Beberok A. Wrześniok D. Kasperczyk J. (2021). Single- versus dual-targeted nanoparticles with folic acid and biotin for anticancer drug delivery. Pharmaceutics 13, 326. 10.3390/pharmaceutics13030326 33802531 PMC8001342

[B12] Kim H. Mondal S. Jang B. Manivasagan P. Moorthy M. S. Oh J. (2018). Biomimetic synthesis of metal–hydroxyapatite (Au-HAp, Ag-HAp, Au-Ag-HAp): Structural analysis, spectroscopic characterization and biomedical application. Ceram. Int. 44, 20490–20500. 10.1016/j.ceramint.2018.08.045

[B13] Kumar R. Shin W. S. Sunwoo K. Kim W. Y. Koo S. Bhuniya S. (2015). Small conjugate-based theranostic agents: An encouraging approach for cancer therapy. Chem. Soc. Rev. 44, 6670–6683. 10.1039/c5cs00224a 26118960

[B14] Li P. Liu Y. Liu W. Li G. Tang Q. Zhang Q. (2019). IR-783 inhibits breast cancer cell proliferation and migration by inducing mitochondrial fission. Int. J. Oncol. 55, 415–424. 10.3892/ijo.2019.4821 31173174 PMC6615916

[B15] Liang S. Liao G. Zhu W. Zhang L. (2022). Manganese-based hollow nanoplatforms for MR imaging-guided cancer therapies. Biomater. Res. 26, 32. 10.1186/s40824-022-00275-5 35794641 PMC9258146

[B16] Manivasagan P. Hoang G. Santha Moorthy M. Mondal S. Minh Doan V. H. Kim H. (2019a). Chitosan/fucoidan multilayer coating of gold nanorods as highly efficient near-infrared photothermal agents for cancer therapy. Carbohydr. Polym. 211, 360–369. 10.1016/j.carbpol.2019.01.010 30824100

[B17] Manivasagan P. Jun S. W. Nguyen V. T. Truong N. T. P. Hoang G. Mondal S. (2019b). A multifunctional near-infrared laser-triggered drug delivery system using folic acid conjugated chitosan oligosaccharide encapsulated gold nanorods for targeted chemo-photothermal therapy. J. Mat. Chem. B 7, 3811–3825. 10.1039/c8tb02823k

[B18] Mondal S. Dorozhkin S. V. Pal U. (2018). Recent progress on fabrication and drug delivery applications of nanostructured hydroxyapatite. Wiley Interdiscip. Rev. Nanomed. Nanobiotechnol. 10, e1504. 10.1002/wnan.1504 29171173

[B19] Mondal S. Hoang G. Manivasagan P. Moorthy M. S. Kim H. H. Vy Phan T. T. (2019). Comparative characterization of biogenic and chemical synthesized hydroxyapatite biomaterials for potential biomedical application. Mater. Chem. Phys. 228, 344–356. 10.1016/j.matchemphys.2019.02.021

[B20] Mondal S. Montaño-Priede J. L. Nguyen V. T. Park S. Choi J. Doan V. H. M. (2022). Computational analysis of drug free silver triangular nanoprism theranostic probe plasmonic behavior for *in-situ* tumor imaging and photothermal therapy. J. Adv. Res. 41, 23–38. 10.1016/j.jare.2022.02.006 36328751 PMC9637560

[B21] Neelgund G. M. Oki A. R. (2016). Influence of carbon nanotubes and graphene nanosheets on photothermal effect of hydroxyapatite. J. Colloid Interface Sci. 484, 135–145. 10.1016/j.jcis.2016.07.078 27599382 PMC5319817

[B22] Park S. Choi J. Mondal S. Vo T. M. T. Pham V. H. Lee H. (2022). The impact of Cu(II) ions doping in nanostructured hydroxyapatite powder: A finite element modelling study for physico-mechanical and biological property evaluation. Adv. Powder Technol. 33, 103405. 10.1016/j.apt.2021.103405

[B23] Phan T. T. V. Bui N. Q. Cho S.-W. Bharathiraja S. Manivasagan P. Moorthy M. S. (2018). Photoacoustic imaging-guided photothermal therapy with tumor-targeting HA-FeOOH@PPy nanorods. Sci. Rep. 8, 8809. 10.1038/s41598-018-27204-8 29891947 PMC5995888

[B24] Prasanna A. Venkatasubbu G. D. (2018). Sustained release of amoxicillin from hydroxyapatite nanocomposite for bone infections. Prog. Biomater. 7, 289–296. 10.1007/s40204-018-0103-4 30478795 PMC6304176

[B25] Qi Y. Qian Z. Yuan W. Li Z. (2021). Injectable and self-healing nanocomposite hydrogel loading needle-like nano-hydroxyapatite and graphene oxide for synergistic tumour proliferation inhibition and photothermal therapy. J. Mat. Chem. B 9, 9734–9743. 10.1039/d1tb01753e 34787633

[B26] Roma-Rodrigues C. Rivas-García L. Baptista P. V. Fernandes A. R. (2020). Gene therapy in cancer treatment: Why go nano? Pharmaceutics 12, 233. 10.3390/pharmaceutics12030233 32151052 PMC7150812

[B27] Rosenblum D. Joshi N. Tao W. Karp J. M. Peer D. (2018). Progress and challenges towards targeted delivery of cancer therapeutics. Nat. Commun. 9, 1410. 10.1038/s41467-018-03705-y 29650952 PMC5897557

[B28] Sabharanjak S. Mayor S. (2004). Folate receptor endocytosis and trafficking. Adv. Drug Deliv. Rev. 56, 1099–1109. 10.1016/j.addr.2004.01.010 15094209

[B29] Santha Moorthy M. Hoang G. Subramanian B. Bui N. Q. Panchanathan M. Mondal S. (2018). Prussian blue decorated mesoporous silica hybrid nanocarriers for photoacoustic imaging-guided synergistic chemo-photothermal combination therapy. J. Mat. Chem. B 6, 5220–5233. 10.1039/c8tb01214h 32254759

[B30] Santhamoorthy M. Thirupathi K. Periyasamy T. Thirumalai D. Ramkumar V. Kim S-C. (2021). Ethidium bromide-bridged mesoporous silica hybrid nanocarriers for fluorescence cell imaging and drug delivery applications. New J. Chem. 45, 20641–20648. 10.1039/d1nj03520g

[B31] Senapati S. Mahanta A. K. Kumar S. Maiti P. (2018). Controlled drug delivery vehicles for cancer treatment and their performance. Signal Transduct. Target. Ther. 3, 7. 10.1038/s41392-017-0004-3 29560283 PMC5854578

[B32] Shanmugam V. Selvakumar S. Yeh C.-S. (2014). Near-infrared light-responsive nanomaterials in cancer therapeutics. Chem. Soc. Rev. 43, 6254–6287. 10.1039/c4cs00011k 24811160

[B33] Shi Z. Zhou Y. Fan T. Lin Y. Zhang H. Mei L. (2020). Inorganic nano-carriers based smart drug delivery systems for tumor therapy. Smart Mater. Med. 1, 32–47. 10.1016/j.smaim.2020.05.002

[B34] Tao B. Lin C. Guo A. Yu Y. Qin X. Li K. (2021). Fabrication of copper ions-substituted hydroxyapatite/polydopamine nanocomposites with high antibacterial and angiogenesis effects for promoting infected wound healing. J. Industrial Eng. Chem. 104, 345–355. 10.1016/j.jiec.2021.08.035

[B35] Terentyuk G. S. Akchurin G. G. Maksimova I. L. Maslyakova G. N. Khlebtsov N. G. Tuchin V. V. (2010). “Cancer laser thermotherapy mediated by plasmonic nanoparticles,” in Handbook of photonics for biomedical science (Florida, United States: CRC Press), 799–834.

[B36] Vinoth Kumar K. C. Jani Subha T. Ahila K. G. Ravindran B. Chang S. W. Mahmoud A. H. (2021). Spectral characterization of hydroxyapatite extracted from black sumatra and fighting cock bone samples: A comparative analysis. Saudi J. Biol. Sci. 28, 840–846. 10.1016/j.sjbs.2020.11.020 33424374 PMC7785448

[B37] Wang Y. Du W. Zhang T. Zhu Y. Ni Y. Wang C. (2020). A self-evaluating photothermal therapeutic nanoparticle. ACS Nano 14, 9585–9593. 10.1021/acsnano.9b10144 32806081

[B38] Wang Y. Wu W. Liu J. Manghnani P. N. Hu F. Ma D. (2019). Cancer-cell-activated photodynamic therapy assisted by Cu(II)-Based metal–organic framework. ACS Nano 13, 6879–6890. 10.1021/acsnano.9b01665 31194910

[B39] Wu X. Suo Y. Shi H. Liu R. Wu F. Wang T. (2020). Deep-tissue photothermal therapy using laser illumination at NIR-IIa window. Nanomicro. Lett. 12, 38. 10.1007/s40820-020-0378-6 34138257 PMC7770864

[B40] Yilmaz B. Evis Z. (2014). Raman spectroscopy investigation of nano hydroxyapatite doped with yttrium and fluoride ions. Spectrosc. Lett. 47, 24–29. 10.1080/00387010.2013.778296

