# Supplementary material for: Biodegradable manganese-doped hydroxyapatite antitumor adjuvant as a promising photo-therapeutic for cancer treatment
Source: Front Mol Biosci. 2022 Nov 23;9:1085458. doi: 10.3389/fmolb.2022.1085458 (PMC9726924; doi:10.3389/fmolb.2022.1085458)
Supplement: Supplementary file 1 [file DataSheet1.docx]

**Supplementary Data**

**Biodegradable Manganese-Doped Hydroxyapatite Antitumor Adjuvant as**

**A promising Photo-Therapeutic for Cancer Treatment**

### Materials characterizations

### The phase purity and crystallinity of the synthesized Mnx-HAp nanoparticles were investigated using X-ray diffraction (XRD, Bruker AXN) analysis. The functional groups were measured using a JASCO FTIR 4100 equipped with Fourier-transform infrared spectroscopy (FTIR). A 532 nm excitation source laser was used to measure the Raman spectra of the JASCO (NRS-5100) instrument. To examine the morphology and ultrastructure, a field emission transmission electron microscope (FE-TEM, JEOL JEM-2100F) was employed. Thermogravimetric and differential thermal analysis (TG-DTA, Perkin Elmer Pyris Diamond) was carried out to examine the thermal stability and phase behavior at a heating rate of 10 °C per minute in an inert atmosphere. Using a UV-vis Diffuse Reflectance Spectrometer (UV-vis-DRS, JASCO V-670), the samples' absorbance and reflectance properties were determined. The average surface area and pore size of the nanoparticles were measured using the Brunauer-Emmett-Teller (BET, Quantachrome Autosorb-iQ) technique.

**Flow cytometry analysis of cell death**

The cells were subjected to 808 nm laser irradiation at 0.75 W. cm^2^ for 7 min after being cultured with varied Mn concentration Mn-HAp nanoparticles for 6 hours. The control group and photothermally treated cells underwent a 6-hour CO_2_ incubator incubation at 37 °C. Then, the live and dead cells were collected, washed with cold PBS, and stained using a FITC-Annexin V Apoptosis kit, per the manufacturer's instructions (BD Pharmingen, San Jose, CA, USA). Following the manufacturer's instructions, flow cytometry (BD Biosciences, CA, USA) was used to evaluate cell death. FlowJo was used to gather and analyze the final results (Ashland, OR, USA). A BD FACSVerse flow cytometer was used for the experimental study using flow cytometry.


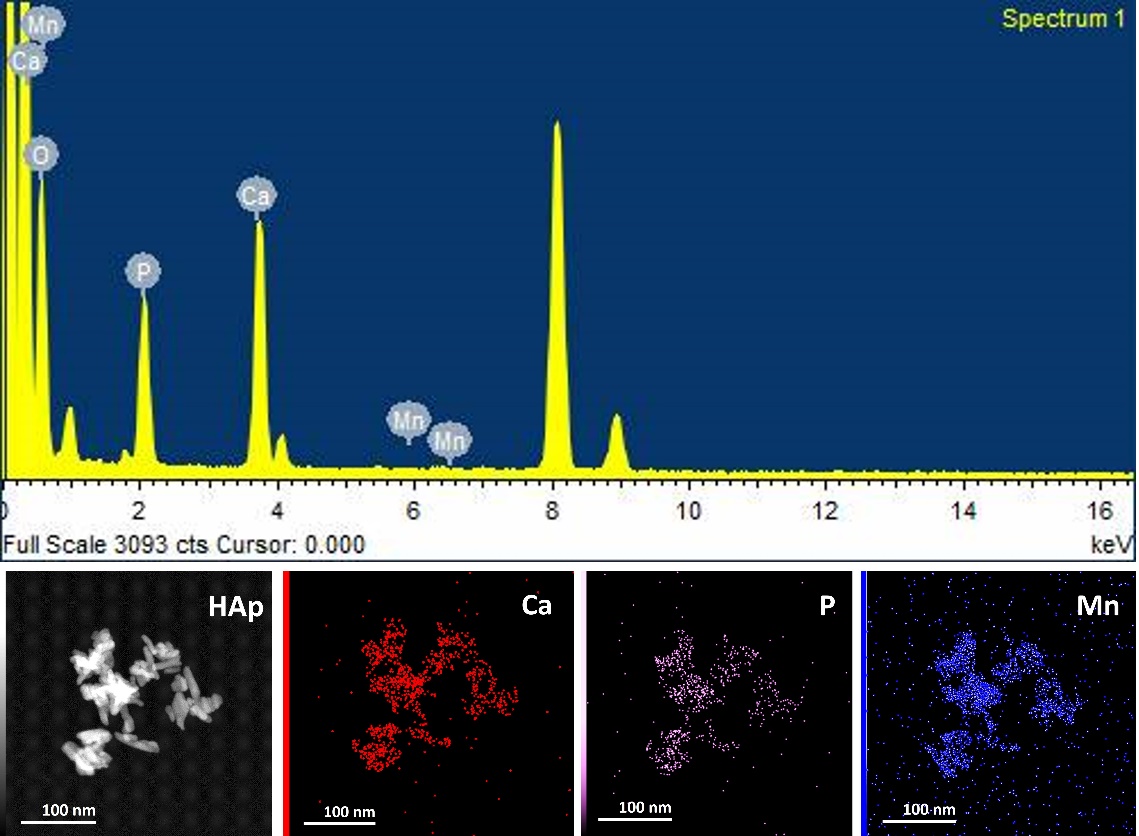


**Figure S1.** Energy dispersive X-ray spectroscopy (EDS) analysis of 2.0 mol.% Mn-HAp. The EDS analysis confirms the presence of Ca, P, and Mn.

**Table S1.** *In vivo* study model with five divided BALB/c female nude mice groups

| Groups | Treatment | Total  animals | Experimental conditions |
| --- | --- | --- | --- |
| I | Control (without tumor) | 4 | Suspension volume: 100 µL  Duration of irradiation: 7 min  Power density: 0.75 W∙cm^-2^  Wavelength: 808 nm  Cancer cells: MDA-MB-231 |
| II | Control (with tumor) | 4 |  |
| III | Only laser no nanoparticles | 4 |  |
| IV | 2.0 mol.% Mn-HAp/FA-IR-783 (no laser) | 4 |  |
| V | 2.0 mol.% Mn-HAp/FA-IR-783 + Laser | 4 |  |
| Total of animals studied | | 20 |  |


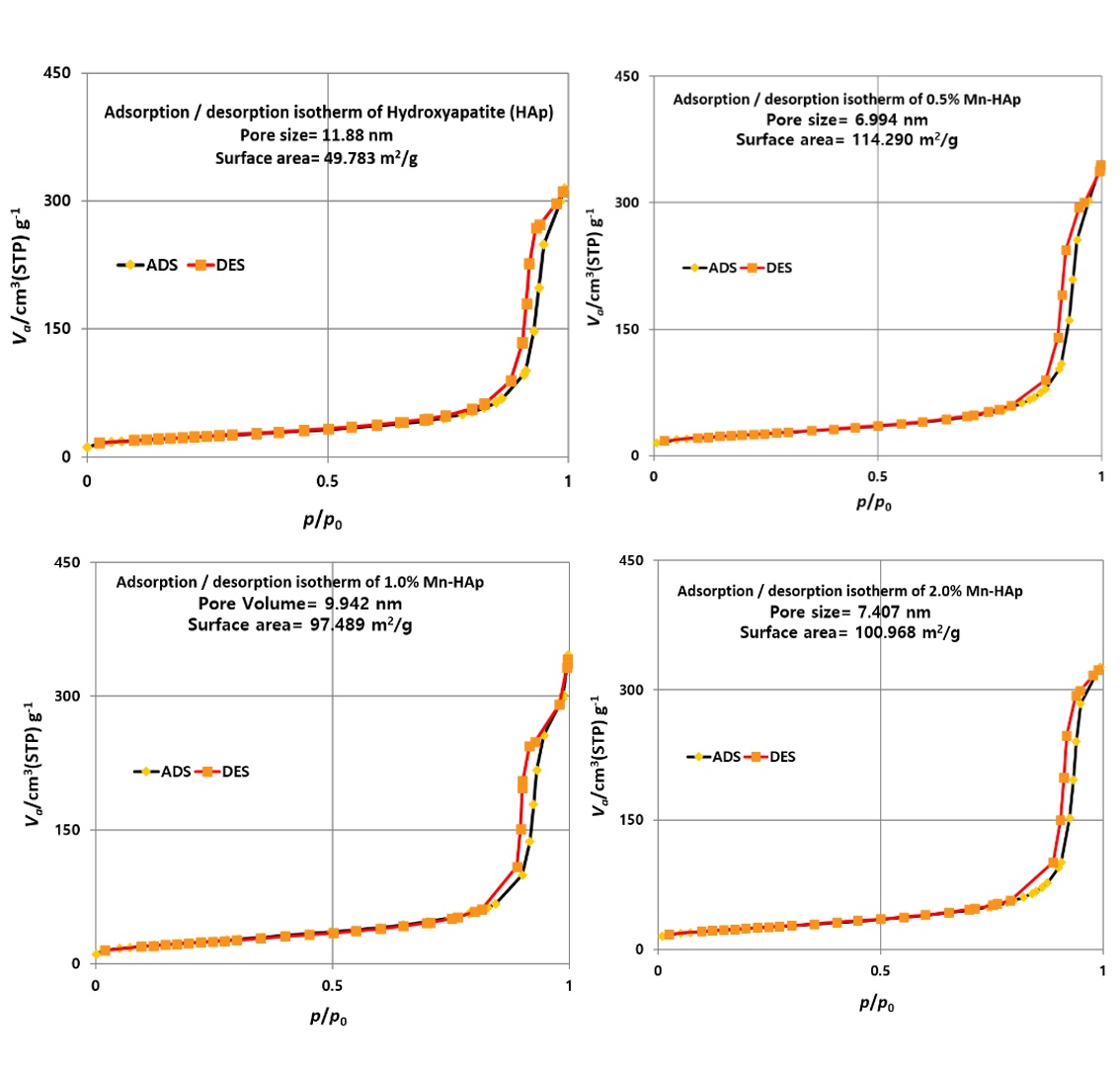
 **BET-Surface area analysis:**

**Figure S2.** BET-surface area analysis of HAp and Mnx-HAp/FA-IR-783 (x = 0.5, 1.0, and 2.0 mol%) nanoparticles.

**Tumor size and volume measurement**

The tumor size and mice weight were measured by digital slide calipers and weight balance. The tumor volumes (*V*) were calculated by the following equation:

$$V=\frac{\pi\times L W^{2}}{6}$$

Here, *V* denotes volume, *L* stands for length, and *W* represents the width of the tumor.

**Photothermal conversion efficiency**

The photothermal conversion efficiency of the 2.0 mol.% Mn-HAp/FA-IR-783 can be calculated as (Hessel et al., 2011):

$\eta=\frac{hS (T_{\max}-T_{\mathrm{env}}{) -Q_{\mathrm{dis}}}}{I(1-10{}^{-A808})}$ (1)

where *h* represents the heat transfer coefficient, *S* is the surface area of the container, and *hS* is obtained from Figure S3b. The maximum steady-state temperature (*T*_max_) of the solution of the 2.0 mol.% Mn-HAp/FA-IR-783 and the environment temperature (*T*_env_) was 26.3 °C. The laser power density, or irradiance *I,* is 0.75 W∙cm^-2^. An 808 is the absorbance of the 2.0 mol.% Mn-HAp/FA-IR-783 nanoparticles at 808 nm. *Q*_dis_ expresses heat dissipated from the light absorbed by the solvent and container. The calculated photothermal conversion efficiency of 2.0 mol.% Mn-HAp/FA-IR-783 to be *η≈* 31.2%.


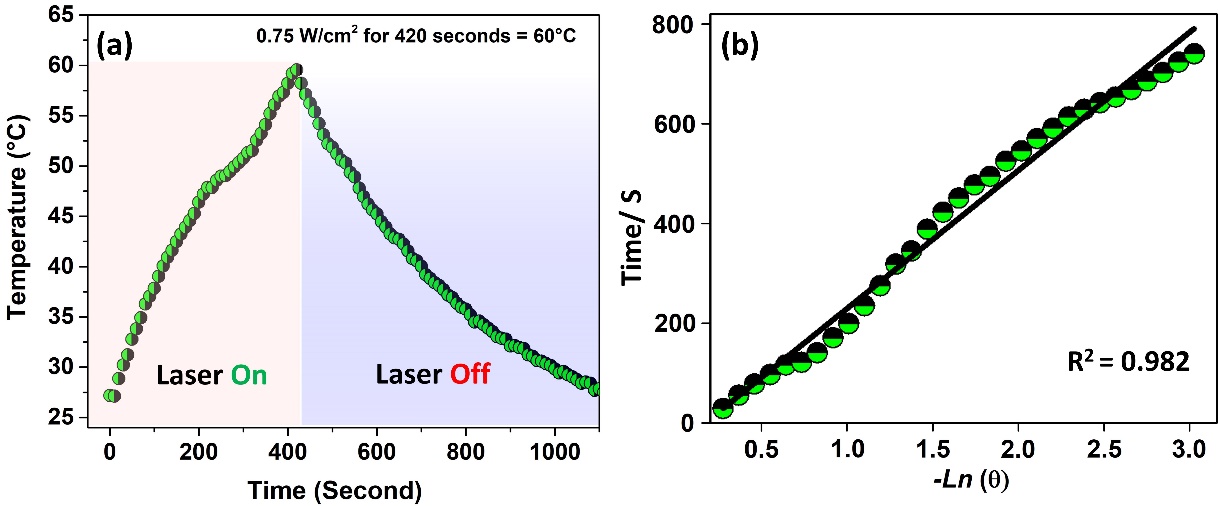


**Figure S3. (a)** Photothermal effect of the irradiation of the aqueous solution of 2.0 mol% Mn-HAp/FA-IR-783 nanoparticles with the NIR laser (808 nm, 0.750 W∙cm^−2^, 420 s). **(b)** Time constant for heat transfer from the system is determined to be $\tau_{s}$ = 119 s by applying the linear time data from the cooling period (after 420 s) versus negative natural logarithm of driving force temperature, which is obtained from the cooling stage of Figure S3b.

**References**

Hessel, C.M., Pattani, V.P., Rasch, M., Panthani, M.G., Koo, B., Tunnell, J.W., and Korgel, B.A. (2011). Copper selenide nanocrystals for photothermal therapy. *Nano letters* 11**,** 2560-2566.

Park, S., Choi, J., Mondal, S., Vo, T.M.T., Pham, V.H., Lee, H., Nam, S.Y., Kim, C.-S., and Oh, J. (2022). The impact of Cu(II) ions doping in nanostructured hydroxyapatite powder: A finite element modelling study for physico-mechanical and biological property evaluation. *Advanced Powder Technology* 33**,** 103405.
